# Book Review

**Published:** 1905-03

**Authors:** 


**Transactions of the Medical Society of the State of New York.** 1904.—This volume, of over 500 pages, contains a large number of original communications, which ought to be very stimulating and cheerful in their effects, inasmuch as the most important of them are grouped together under the term "Symposium." This fashionable American term is applied to any one of the following descriptions given by Ogilvie's *Imperial Dictionary:* a drinking party, a drinking together, a merry feast, a convivial meeting. The one connecting link is the Greek word *pino*, to drink. The subject of the first series of papers is appropriate, viz. Diabetes. Five papers on Diabetes, on the pathology, the physiological chemistry, the medical treatment, the general management, and the occurrence of Diabetic and Non-diabetic Glycosuria in the same individual, form a very instructive group of papers, which produce a kind of polydipsia for more. But here the series terminates, and is merged into another symposium—this time a series of three papers on Nephritis, a condition in which, according to Van Noorden, the fluids should be strictly limited. Other symposia follow: two papers on Abdominal Pain, five on Typhoid Fever, and four on the Roentgen Ray. This method of grouping papers on the same subject is excellent; but why should they be all associated with a term implying thirst and conviviality? Many other excellent papers are not so designated, and are worthy of careful study.

**Studies from the Rockefeller Institute for Medical Research.** Reprints. Vol. I. 1904.—This book consists of a series of reprints of papers which have mostly appeared in other journals, and are here collected into a single volume. They owe their origin to the fact that, pending the complete organisation of the Rockefeller Institute for Medical Research, the directors of the institute made a series of grants to workers in various laboratories in the United States. According to the original intention of the founder of the institute, these papers consist of original researches into the causes and treatment of disease. They are mostly on bacteriological subjects. Attention may be directed to the series of articles on the bacteriology of acute dysentery, the relation of the dysentery bacillus to infantile diarrhœa, and the possibility of a serum-therapy against dysentery. There are also several papers dealing with the actions of adrenalin, both on the vessels and on the pancreas. All the contributions attain a high level, and as they all embody original research on subjects in which advance is being made in medicine, they deserve careful study. Reprints. Vol. II. 1904.—This interesting collection of papers on various subjects gives an excellent impression of the valuable work done by the Fellows of the Rockefeller Institute in the United States, Canada, and Germany. Fully one-third of the volume is devoted to the elaborate researches into the diarrhœal diseases of infants conducted under the direction of Drs. Holt and Flexner. Under them a large group of workers have investigated the clinical and bacteriological problems connected with "summer diarrhœa" in a systematic manner, the conclusions arrived at being set forth in two lucid and masterly papers by the directors of the scheme. In order to afford critics full data as to the observations on which these conclusions are based the reports of the various workers are printed *in extenso*. The Flexner type of the dysentery bacillus which closely resembles that isolated by Shiga in Japan, was proved to be the causal agent in many cases of "summer diarrhœa" in infants occurring in the eastern cities of the United States. That this bacillus is widely distributed has been shown by the researches of Kruse in Germany and Eyre in England, but whether the "summer diarrhœa" of infants in this country is caused by it either wholly or in part has not been determined. Serum treatment did not give in America any very startling results; this no doubt is due to the fact that the disease is not wholly due to toxæmia, as Dr. Holt points out, many of the symptoms being due to local disturbances of the digestive tract. In a supplementary paper by Dr. Martha Wollstein the dysentery bacillus was shown to be but rarely present in the discharges of children not suffering from disease of gastro-intestinal origin. Drs. Park and Holt's paper upon the results of feeding infants on different kinds of pure and impure milk, which is now familiar to all interested in the diseases of children, is reprinted in this volume. Their conclusions are too well known to call for fresh comment, and are as interesting from the point of view of sociology and public health as from that of pure bacteriology. From the latter standpoint there are several papers of interest among the reprints. Ford writes on the human intestinal flora in great detail; a paper by Wolbach and Ernst on the morphology of the tubercle bacillus is of interest in connection with Marmorek's recently-described experiments, while Perkins, in a carefully-described series of observations, suggests a method of classification for the group of bacteria represented by the bacillus mucosus capsulatus. The other contributions are: Preston Keyes on the isolation of lecithins of snake poison (cobra venom); Sweet on the hæmolytic, bacteriolytic, and bactericidal reactions of the blood of animals in which artificial glycosuria and diabetes mellitus had been produced; Ehrlich and Marshall on the complementophil groups of amboceptors; Novy and McNeal on the cultivation of the trypanosoma of the Tsetse fly disease; and Opie on the relation of eosinophile cells to nutrition—an exhaustive review and investigation which has already appeared in the American *Journal of Medical Research.*

**The Student's Handbook of Surgical Operations.** By Sir Frederick Treves, Bart., K.C.V.O., C.B. New Edition. Revised by the Author and Jonathan Hutchinson, Jun. Pp. xii., 486. London: Cassell and Co. Limited. 1904.—The second edition of this book is an outcome of a revised edition of the larger manual by the same authors recently published. As it is abridged from this, the alterations are naturally the same in the two works. These concern chiefly the chapters on abdominal surgery, which are now very much out of date in the old edition. Specially may be noted amplified accounts of intestinal suture, operations on the stomach, on the gall bladder and ducts, and a very much more detailed description of the radical cure of hernia. For some reason no mention is made of hysterectomy, which is rather a pity, as otherwise the book is wonderfully full for its size. It has not been increased in size by the alterations and additions, and still remains a *multum in parvo* of operative surgery.

**Handbook of Diseases of the Ear.** By Richard Lake. Second Edition. Pp. x., 242. London: Baillière, Tindall & Cox. 1904.—The early appearance of a second edition of this work testifies to its popularity. With the addition of only ten pages several improvements have been made over the last edition, and many printers' errors and ambiguous sentences have been corrected. A section has been devoted to the treatment of chronic suppuration in the middle ear apart from that of its complications, which somehow had been omitted in the first edition, and three pages have been added to the short chapter on diseases of the internal ear. It is the best book of its size published in this country on diseases of the ear.

**Cancer: Its Causation and Curability without Operation.** By Robert Bell, M.D. Pp. viii., 271. London: Baillière, Tindall & Cox. 1903.—Dr. Bell claims that by purely medical means he is able to cure cancer, and he says "it (*i.e.* cancer) only *appears* to have no remedy to those who have not attempted to employ the remedies which I know exist." The remedies referred to are chiefly extracts of the thyroid and mammary glands. A few instances of cures are quoted, and in some of these cases curetting of the affected uterus was performed as a preliminary measure, but unfortunately the microscopic reports have been omitted, and as far as we are able to gather, in not a single instance where success is claimed was the diagnosis of cancer confirmed by pathological methods! The arguments are incomplete and far from convincing, and the language in which they are couched is poor and in many places ungrammatical.

**Golden Rules of Anæsthesia.** By R. J. Probyn-Williams, M.D. Pp. 67. Bristol: John Wright & Co. [n.d.].—Dr. Probyn-Williams has made a clever selection of the most important facts to be borne in mind by administrators of anæsthetics. Students will find this book a great help as a guide to them while acquiring a practical knowledge of the subject. We notice one mistake which has evidently been overlooked. On page 44 the dose of ethyl chloride, as a substitute for gas before ether, is given as "three or four minims" instead of "three or four cubic centimetres."

**Medical Officer of Health, Annual Report of, for the City and County of Bristol.** 1903.—This report needs little comment, as it is for the most part the detailed account of which we gave an abridged abstract in a paper by the Medical Officer of Health in March last (*vide* page 55). The report is printed by order of the Health Committee, and it gives a very full account of all matters of interest relating to the sickness and mortality of the year 1903. We observe that the Council has approved of the initiation of a voluntary system of notification of phthisis, but that the arrangements for carrying out the scheme are not yet completed. The distribution of leaflets throughout the city has been undertaken by the members of the St. John Ambulance Brigade, who will also provide Dettweiler's sputum flasks, free of cost, to consumptive patients who are unable to afford to buy them.

**The Journal of the Röntgen Society.** Vol. I., No. 1. Printed for the Röntgen Society by Wertheimer, Lea & Co., Finsbury, E.C.—We congratulate the Röntgen Society on at length possessing a journal of its own. The new journal is of a most convenient size, well printed, and the illustrations far above the average; in fact, the reproduction of the photograph of Professor Silvanus Thompson, the first President of the Society, which is appropriately placed at the commencement of the volume, is quite a work of art. If the succeeding numbers of the journal fulfil the promise of the first, the *Journal of the Röntgen Society* will be a notable addition to our periodical literature.

**Notes on Assouan, Egypt.** By G. Dundas Edwards, M.A. Pp. 36. London: John Bale, Sons & Danielsson. 1904.—Any intending travellers to Egypt would do well to peruse this booklet of thirty-six pages. Although few go so far from Cairo, the attractions described by Mr. Edwards may induce visitors to extend their travels towards the south, in order that they may enjoy the absolutely uncontaminated and dry air of the Libyan desert. Assouan is situated three miles north of the famous dam, about 580 miles from Cairo. It can be reached by train in about twenty-three hours, but the voyage up the Nile by tourist boat is certainly the more agreeable route. The features of the climate are: (1) the purity of the air; (2) the large amount of sunshine; (3) the warmth; (4) the dryness of the air. The aseptic air of the desert appears to have a peculiarly beneficial action on inflamed mucous membranes, and we learn that a man cannot drink the same amount of whisky in Khartoum that he does in Glasgow. This rule applies equally to drugs, as the same dose of strychnine or arsenic has not the same effect in all climates. We shall look to Mr. Edwards to give us some further investigations on these interesting questions. He tells us that camel-riding is the most terrible form of exercise ever invented.

**Index-Catalogue of the Library of the Surgeon-General's Office, United States Army.** Second series. Vol. IX.: L—Lyuri. Pp. 872. Washington: Government Printing Office. 1904.—This volume includes 8,706 author-titles, 5,322 subject-titles of separate books and pamphlets, and 31,481 titles of articles in periodicals. The labour of the editors must be great, but they have the satisfaction of knowing that their monumental work is absolutely indispensable to any well-equipped medical library.

**The Diagnosis and Modern Treatment of Pulmonary Consumption.** By ARTHUR LATHAM, M.D. Oxon. Second Edition. Pp. vii., 224. London: Baillière, Tindall and Cox. 1905.—The utility of this prize essay is shown by the fact that the first impression has been so soon exhausted. This second edition has been very slightly enlarged, and it has been improved by a few useful additions and amplifications. It continues to be one of the best of guides to sanatorium life and treatment.

**The "Nauheim" Treatment of Chronic Diseases of the Heart.** By LESLIE THORNE THORNE, M.D. Pp. 53. London: Baillière, Tindall and Cox. 1904.—This little book is designed to give those wishing to practice the Nauheim treatment in England some knowledge of how to prepare the baths, and the method of their use. An outline is also given of the character of the cases which the author considers suitable for the treatment, and also of those which are unsuitable. The book closes with a few illustrative cases.

**Enlargement of the Prostate, its Treatment and Radical Cure.** By C. MANSELL MOULLIN, M.D. Oxon., F.R.C.S. Third Edition. Pp. xi., 204. London: H. K. Lewis. 1904.—We have carefully perused this, the third edition, and have been impressed with the consistent accuracy and judgment expressed throughout the book. The closing sections, dealing with the results of various methods of treatment, may be specially commended, although the value of the book might be enhanced by illustrations of the various methods of operation. The lack of illustrations is a defect in what is otherwise a well-written and practical work, and one that can be warmly recommended, even if it only be to disclose "the consequences that follow from the indiscriminate use of catheters."

**The Case Against Anti-vivisection.** By STEPHEN PAGET. Pp. 104. London: The Scientific Press, Ltd. 1904.—This is a valuable little book for anyone interested in the vivisection controversy. Apparently the first protests in England against cruel experiments were made in the medical journals—in the *Medical Times and Gazette* in 1858, in the *Lancet* in 1860, and in the *British Medical Journal* in 1861. This small book deals briefly with the literature and arguments of the anti-vivisection societies, and interesting examples are given of the character of their statements. For example, "the object of the sportsman is to kill, the object of the experimenter is to torture." Again, it is stated that vivisectionists support vivisection because it pays them to do so, presumably to prepare serums. There is "money in the business," says the leading anti-vivisectionist journal; "there is milk in the cocoa-nut, and twopence more and up goes the donkey." With regard to curare, which, according to an anti-vivisectionist writer, is "in daily use throughout England," Mr. Stephen Paget found that during 1903 it had been used at Oxford, Cambridge, and Edinburgh only twelve times, and in seven out of those twelve cases it was in pithed frogs. In the remaining five cases it is needless to say that the animals were under A.C.E. or ether.

A companion book, more valuable perhaps, and not less interesting, is *What we Owe to Experiments on Animals*. A brief survey is given of much that has been learnt in physiology, pathology, and pharmacology from experiments upon animals, and how much that is perhaps may not be realised until such a book, which puts the matter concisely before us, is read.

**The Antiseptic.** Vol. I., No. VIII. December, 1904. Madras. —This new exchange is a monthly journal of medicine and surgery, the first of its kind to be published in the Madras Presidency. It consists of nearly forty pages, many of the items being specially-contributed original articles. With these are some well-chosen cases of interest, leading articles, and reviews from contemporaries.

